# Stopping cells from dividing

**DOI:** 10.18632/aging.100938

**Published:** 2016-03-29

**Authors:** Kurt Engeland

**Affiliations:** Molecular Oncology, Medical School, University of Leipzig, Leipzig, Germany

**Keywords:** cell cycle arrest, p53, DREAM, E2F, CHR site, transcription, pRB, p130, p21 CDK inhibitor

Is there anything closer to the heart of preventing cancer than inhibiting cell division? The quest to identify means to stop cells from proliferating has driven generations of researchers to investigate the mechanisms regulating the cell division cycle. The cell cycle consists of several phases. Progression from one phase to the next depends on the activity of specific proteins. In order to pass the checkpoints between two phases, several classes of proteins are required. These regulators include transcription factors, kinases, phosphatases, and proteins acting through complex formation. The synthesis of these proteins - particularly transcriptional control of their genes - constitutes an important mode to regulate cell division.

A key regulator of the cell cycle is the tumor suppressor p53. It is a transcription factor which functions by activating genes [1]. The cyclin-dependent kinase inhibitor *p21/CDKN1A* gene was the first identified p53 transcriptional target. In addition to genes being activated, many genes become downregulated upon p53 activation. For transcriptional repression by p53 several - often conflicting - mechanisms have been proposed [2]. In fact, there is evidence that p53-mediated downregulation is always indirect [3]. Transcriptional repression by p53 appears to occur without direct contact of p53 to its target genes [3]. With *Cyclin B2* as the first example, a novel pathway was suggested which explains the mechanism of transcriptional repression by p53 [4]. Specifically, this pathway is not initiated by repression but by p53 transcriptionally activating *p21/CDKN1A*. The p21/CDKN1A protein then inhibits Cyclin/CDK kinase complexes (Figure 1). This causes hypophosphorylation of the pRB-related pocket proteins p130 and p107. Their reduced phosphorylation shifts the equilibrium from the activating MMB to the repressing DREAM complex. Binding of DREAM to CHR promoter elements finally downregulates target genes [4]. CDE sites four nucleotides upstream of CHR elements can support binding of DREAM to DNA (Figure 1). Taken together, stimulation of the p53-p21-Cyclin/CDK-DREAM-CDE/CHR pathway causes transcriptional downregulation of target genes after p53 has been activated [4].

**Figure 1:**
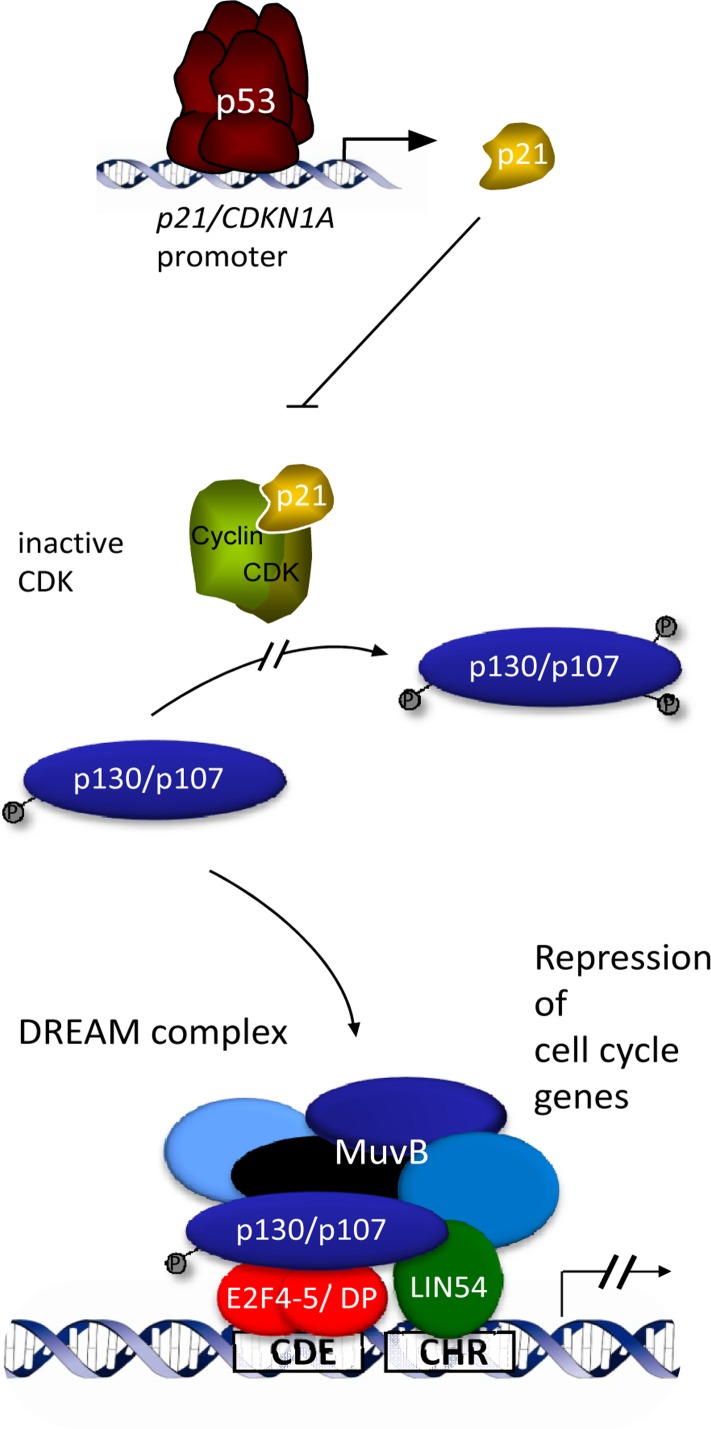
Indirect repression of cell cycle genes by p53

After resolving this pathway for one gene, the main question was whether this indirect mechanism of p53-dependent transcriptional repression applies also to other genes. In a recent report published in *Oncotarget*, three genes important for cell cycle regulation have been shown to be controlled by the p53-p21-Cyclin/CDK-DREAM-CDE/CHR pathway [5]. *Survivin* (*BIRC5*), *CDC25C*, and *PLK1* represent genes that had been previously examined in several reports for their transcriptional control during the cell cycle and in regard to their response to p53 activation [5]. However, incomplete and partially contradicting observations had been published specifically on transcriptional regulation of these genes by p53 [2;5]. The current report resolves these issues and presents a consistent mechanism of indirect transcriptional repression by p53 for *Survivin*, *CDC25C*, and *PLK1* through the p53-DREAM axis [5].

The DREAM complex is not only involved in p53-mediated transcriptional repression. Mammalian DREAM was discovered because of its role in transcriptional repression of cell cycle genes. It is composed of the MuvB core complex, E2F4/DP (or E2F5/DP), and the pRB-related p130 protein (or p107) [6]. Interestingly, MuvB can also form the basis for complexes that activate genes. In such cases, proteins associated with the MuvB core change, switching the function of the resulting complex from repression to activation. Activating MuvB complexes do not contain E2F/DP/pRB-related protein components. Instead, these complexes comprise the transcriptional activators B-MYB or FOXM1 [6]. All combinations of the resulting MuvB-based complexes - DREAM, MMB, and FOXM1-MuvB - require CHR elements in the target promoter for the LIN54 component of MuvB to bind DNA [4].

Transcriptional repression by the p53-p21-Cyclin/CDK-DREAM-CDE/CHR pathway appears to be a general mechanism in cell cycle control [7]. Several genome-wide chromatin immunoprecipitation (ChIP) analyses for DREAM components have been performed to identify DREAM target genes. Bioinformatic analyses of these DREAM ChIP data in combination with genome-wide p53-dependent mRNA expression data and identification of phylogenetically conserved CHR elements yielded a list of more than 200 predicted p53-p21-Cyclin/CDK-DREAM-CDE/CHR pathway targets [7]. The proteins encoded by the genes controlled by the pathway represent regulatory mechanisms such as transcription, guanine nucleotide exchange, chromatin modification, ubiquitination, complex formation, proteolysis, phosphorylation, and dephosphorylation [7]. With the examples *B-MYB (MYBL2), BUB1, CCNA2, CCNB1, CHEK2, MELK, POLD1, RAD18, RAD54L*, *Survivin*, *CDC25C*, *PLK1, CCNB2, PLK4,* and *KIF23,* regulation by the p53-p21-Cyclin/CDK-DREAM-CDE/CHR pathway was validated experimentally [4;5;7]. Taken together, these results suggest that the list of 210 candidates from the bioinformatic analyses represents *bona fide* p53-p21-Cyclin/CDK-DREAM-CDE/CHR pathway target genes [7]. Among the targets are important cell cycle factors such as Aurora kinase A/B, several cyclins, CDC25A/B/C, cyclin-dependent kinase 1 and 2, several kinesins, Polo-like kinases, SUZ12, and TTK [7]. Essentially all aspects of cell cycle regulation are represented with proteins involved for example in cell cycle checkpoint control, DNA replication, mitotic spindle assembly, and DNA repair [7]. Thus, it appears that the p53-p21-Cyclin/CDK-DREAM-CDE/CHR pathway has a general function in stopping cells from dividing.

## References

[1] Vousden KH (2009). Cell.

[2] Böhlig L (2011). J. Biomed. Biotechnol.

[3] Fischer M (2014). Cell Cycle.

[4] Quaas M (2012). Cell Cycle.

[5] Fischer M (2015). Oncotarget.

[6] Sadasivam S (2013). Nat. Rev. Cancer.

[7] Fischer M (2016). Nucleic Acids Res.

